# Constrained Optimization of Average Arrival Time via a Probabilistic Approach to Transport Reliability

**DOI:** 10.1371/journal.pone.0126137

**Published:** 2015-05-20

**Authors:** Mohammad-Reza Namazi-Rad, Michelle Dunbar, Hadi Ghaderi, Payam Mokhtarian

**Affiliations:** 1 SMART Infrastructure Facility, University of Wollongong, Wollongong, NSW 2522, Australia; 2 National Institute for Applied Statistics Research Australia, University of Wollongong, Wollongong, NSW 2522, Australia; 3 Department of Maritime & Logistics Management, National Centre for Ports & Shipping, Australian Maritime College, University of Tasmania, Launceston TAS 7248, Australia; Beihang University, CHINA

## Abstract

To achieve greater transit-time reduction and improvement in reliability of transport services, there is an increasing need to assist transport planners in understanding the value of punctuality; i.e. the potential improvements, not only to service quality and the consumer but also to the actual profitability of the service. In order for this to be achieved, it is important to understand the network-specific aspects that affect both the ability to decrease transit-time, and the associated cost-benefit of doing so. In this paper, we outline a framework for evaluating the effectiveness of proposed changes to average transit-time, so as to determine the optimal choice of average arrival time subject to desired punctuality levels whilst simultaneously minimizing operational costs. We model the service transit-time variability using a truncated probability density function, and simultaneously compare the trade-off between potential gains and increased service costs, for several commonly employed cost-benefit functions of general form. We formulate this problem as a constrained optimization problem to determine the optimal choice of average transit time, so as to increase the level of service punctuality, whilst simultaneously ensuring a minimum level of cost-benefit to the service operator.

## 1 Introduction

In designing a schedule for real-world transport systems (e.g. buses, trains, container ships or airlines), transport planners typically adopt a tactical-planning approach [1–3]. As such, scheduling decisions are typically performed a few weeks or months prior to the day-of-operations, with only minor changes to the schedule permissible under certain conditions on the day-of-operations. The process usually begins with a choice of route and its corresponding stopping pattern. Once this has been determined, specific vehicles and crew are then assigned to individual routes. Until recently, the primary tenet of schedule planning has been one of cost-cutting and maximizing profit. In order to achieve this, schedulers seek to maximize the efficiency and utility of their existing resources and so often choose to keep the amount of down-time, commonly referred to as slack, as minimal as possible across the network [4–6].

This approach however assumes that all operations will be carried out as planned, and thus is deterministic in design; not accounting for unexpected service interruptions, random fluctuations during peak services or knock-on delay. As reliability of a transport system becomes a crucial decision making element from both transport user and provider perspectives, such an assumption is no longer applicable to the majority of real-world networks. The underlying characteristics of an optimal transit service is the ability of the service to provide effective standards for management and operation, in such a way as to ensure that negative customer perceptions associated with quality-of-service, (such as unreliability and late-arrival,) are minimized as much as possible. These are indeed key factors for determining the customer’s modal choice, transport carrier, and thus the profits of both service providers and users [3, 7–9].

As discussed by [5], the two key elements in ensuring the quality and reliability of transport services are: *i*) *timing*: that is, scheduled departure and arrival time for the specified service and *ii*) *punctuality*: the ability of the service to remain as close as possible to the planned schedule. Consequently, as user demand for transport services continues to grow, there is a pressing need for schedule planners to design schedules in such a way as to incorporate and plan for operational uncertainty. This trade-off between variability of punctuality level, transit times and travel costs is discussed in the literatures [10–13].

Due to the growing discrepancy between planned costs and realized costs, many transport operators must now accommodate for both rising service costs and costs resulting from unforeseen disruption and subsequent schedule recovery in addition to accommodating disrupted passengers. Care must be taken however to avoid simply padding the schedule with slack, indiscriminately, as this is not only costly and inefficient, but it also ignores the behavioral response of operators who are provided with additional time as a buffer and display a tendency to take longer to complete the required task [11]. This in turn affects customer perceptions of quality-of-service as well as the satisfaction of external clients. In contrast, a reliable transport service ensures commercial viability of the system in long-term [14].

There is therefore a need to design schedules in such a way as to allocate additional slack to the operations in which it is needed the most; whilst seeking to simultaneously minimize travel time or a range of alternative objectives. For example, the objective of a passenger transport network may be to maximize on-time performance (OTP) [15–17], whilst a freight transport network may seek to maximize throughput, or make-span. In addition, there may be a variety of costs, varying with time-of-day, or time-of-arrival which influence the choice of arrival time or stopping pattern. Consequently, different transport operators may assume a variety of different performance functions according to their specific context. Thus, a one-size-fits-all approach would generally be insufficient in capturing the complexities involved with modern transportation systems, and their cost trade-offs.

With the advent of just-in-time production for many real-world supply chains and the growing population in urban centers, transport operators require a methodology for designing more reliable, responsive and network specific schedules that are both profitable for the operator whilst suiting the needs of their customers. In this paper, we propose a framework by which operators may examine *a priori*; the trade-off between improvements in punctuality and operating cost. We provide an analysis of the effectiveness and potential gains that an operator may achieve through minor adjustments to the transit-time distribution, comparing this with the associated cost trade-offs for a number of different real-world scenarios.

The remainder of this paper is laid out as follows. In Section 2 we analyze the improvements in punctuality that may be achieved using a truncated Poisson distribution. In Section 3, we perform a cost-benefit analysis of such adjustments for three potential network scenarios, defining the cost-benefit curves under each scenario. In Section 4, we investigate the cost of delays and propose a penalty function to be used in conjunction with the cost-benefit curve for each scenario. Finally, we combine these measures in Section 5 for analyzing the relationship between cost-benefit, cost of delay and punctuality for each of these scenarios.

## 2 Transit Time Distributions

In this section, we investigate the relationship between transit-time modification and the resulting level of punctuality. As mentioned earlier, service punctuality is a key driver in determining customer perceptions of quality-of-service, and according to [18], punctuality is often used as a reliability performance measure for transport systems.

Intuitively, negative deviations (delays) from the scheduled timetable are associated with poor levels of punctuality. Punctuality is often used as a discrete measurement related to a predefined level of accepted deviation [19]. Many transport operators such as airlines and train services have their own definition of what constitutes OTP for their respective networks. For example, airlines consider any arrival within 15 minutes of the scheduled arrival time to be ‘on time’, whilst smaller delays, such as 5–10 minutes are considered acceptable for passenger train services. One of the reasons for re-defining the meaning of OTP is due to the natural desire of operators to avoid additional costs in keeping to a schedule that is often “over optimized”—that is, a schedule for which the effects of potential disruption have been ignored, and are often brittle in practice. While this may appear to disguise the effects of delay to the customer, even small deviations such as these, can have dramatic ramifications throughout the network, in the form of knock-on delays and canceled services, and even greater recovery costs. Furthermore, an analysis of the effects of even minor changes to the transit-time on punctuality level may be of great benefit in the decision making process for planners, as well as a knowledge of which services are the key-players in keeping the entire timetable to schedule.

For transport operators to sufficiently understand punctuality for their specific network, an accurate set of data for each service is essential. As we are examining a potential set of network scenarios, we adopt a more general modeling framework and present a statistical probabilistic approach for investigating the impact of transit time reduction on service punctuality. To define such a model, we assumed *μ* to denote the mean (average) transit time and *t*_*s*_ to denote the scheduled transit arrival time. The maximum transit arrival time is denoted by *t*_*Max*_, and corresponds with the maximum allowable deviation permissible without having to cancel down-the-line services. In addition, we consider the minimum transit arrival time (denoted by *t*_*Min*_ < *t*_*s*_) to represent the earliest time by which a service may arrive without being refused access at the destination (ie. gate/station/port). With these assumptions, we may now model the transit-time distribution as a two-sided truncated Poisson distribution. The idea of using truncated distributions for transportation modeling is discussed by [20–22]. Here, we assume *X* to define a discrete random variable with probability density function *f*(*x*) = *Pr*(*X* = *x*), and the two-side(*t*_*Min*_, *t*_*Max*_)-truncated function as:

$$\begin{array}{c} \begin{array}{c} g\left(x\right)=f\left(x|t_{Min}\leq X\leq t_{Max}\right)=\frac{f^{*}\left(x\right)}{Pr\left(X\leq t_{Max}\right)-Pr\left(X<t_{Min}\right)}\;. \end{array} \end{array}$$ (1)

Note that, f*(*x*) = *f*(*x*) for all *t*_*Min*_ ≤ *X* ≤ *t*_*Max*_. Here, *g*(*X*) has the same support as f*(*x*). The function *g*(*x*) is a probability density function with *t*_*Min*_ ≤ *X* ≤ *t*_*Max*_ support and the total probability over the support is equal to ‘1’ as in: [23]

$$\begin{array}{c} \begin{array}{c} \sum_{x=t_{Min}}^{t_{Max}}g\left(x\right)=\frac{1}{Pr\left(X\leq t_{Max}\right)-Pr\left(X<t_{Min}\right)}\sum_{x=t_{Min}}^{t_{Max}}f^{*}\left(x\right)=1\;. \end{array} \end{array}$$ (2)

In transport service scheduling, a service is considered punctual if the transit time is less than a certain punctual-time (denoted by *t*_*p*_). The cumulative distribution function of the two-side (*t*_*Min*_, *t*_*Max*_) truncated distribution with parameter *θ*, representing the probability function for the service to be classified as reliable/punctual, is as follows:

$$\begin{array}{c} G\left(x|\theta \right)=\sum_{x=t_{Min}}^{t_{Max}}g\left(x|\theta \right). \end{array}$$ (3)

Some researchers such as [11] have used the Beta distribution to model arrival distributions in transportation and rescaled and translated the parameters so as to be defined on the interval bounded in between *t*_*Min*_ and *t*_*Max*_. However, using such a technique, the probability of arrival time close to *t*_*Min*_ is very small which seems to be an unrealistic assumption in practice. Alternatively, the assumption that a point-to-point transit time follows a Poisson distribution is also discussed in the literature [24–28]. Here, we assume that transit time denoted by *x* follows a Poisson distribution with parameter *λ*. Then,

$$\begin{array}{c} f\left(x|\lambda \right)=\frac{e^{-\lambda }\lambda^{x}}{x!}\;;\;x=0,1,2,\ldots \end{array}$$ (4)

where, the average transit time is:

$$\begin{array}{c} \mu =E(X)=\lambda \;. \end{array}$$ (5)

Thus, the two-side (*t*_*Min*_, *t*_*Max*_)-truncated Poisson distribution is:

$$\begin{array}{c} g\left(x|\lambda \right)=\frac{1}{Pr\left(X\leq t_{Max}\right)-Pr\left(X<t_{Min}\right)}\times \frac{e^{-\lambda }\lambda^{x}}{x!}\;;\;x\in (t_{Min},t_{Max}]\;. \end{array}$$ (6)

Clearly, transport service providers are interested in increasing the punctuality of their service while this is possible by decreasing the average arrival time by managing the service constraints. By reducing the average transit time, the probability function of punctuality of the service is given by:

$$\begin{array}{c} G\left(x|\lambda_{i}\right)=\sum_{x=t_{Min}}^{t_{p}}g\left(x|\lambda_{i}\right)\;;\;\lambda_{i}\in \left\{\lambda ,\lambda -1,\ldots ,t_{p}\right\},\lambda <t_{p}\;. \end{array}$$ (7)

**Fig 1:**
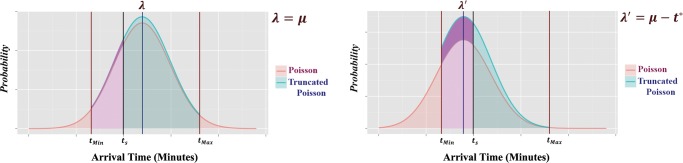
Non-truncated and truncated probability density functions for transit time (the underlying distribution in the colour ‘magenta’).

Fig 1 shows the probability density function (PDF) of the arrival time falling within the punctual interval by marginally reducing the transit time.

In Fig 1 and in the graph presented on the left, *λ* is assumed to denote the average arrival time while the minimum and maximum arrival time are respectively denoted by *t*_*min*_ and *t*_*max*_. While the scheduled transit time is denoted by *t*_*s*_, the probability of the transit time falling between *t*_*min*_ and *t*_*s*_ is equal to the the total area underneath the PDF between these two points shown in the color ‘magenta’. In the graph presented on the right, the average arrival time is decreased by t*. In this case, the probability that the transit time falls between *t*_*min*_ and *t*_*s*_ (shown in the color ‘magenta’) increases. The graphs presented in Fig 1 also show the difference between the area underneath the PDF and the truncated PDF. As shown in Fig 1, the probability of the reliability increases when the average transit time is reduced. Although there is great potential for improving punctuality by decreasing the average arrival time, there will naturally be an associated cost trade-off of doing so. We now examine this cost trade-off in the following section for three different network scenarios.

## 3 Cost-Benefit Analysis

As mentioned above, changes to the average scheduled arrival time will incur additional operational costs. There have been a number of attempts to quantitatively describe these costs associated with changes to the schedule from a service, passenger, life-cycle and environmental standpoint [9, 29–33]. These usually focus on costs associated with either streamlining operations to decrease average transit-time, or the costs associated with arriving after the scheduled arrival time, namely delay costs. Moreover, these functions are usually simplistic in nature, and not tailored to the specific network, as typical cost functions are assumed to be either linear, quadratic or exponential.

In this paper we extend these approaches and define a cost-benefit function for both of these aspects, so as to propose a *composite measure* capturing *more realistic* costs associated with *both* positive and negative deviations from the scheduled arrival time. We begin by proposing three potential cost functions for the case in which the schedule is subject to positive deviations (decrease in average arrival time) for the aircraft scheduling problem. These cost-benefit functions will be described according to *general* input parameters that define their essential properties. Specific values for each of these parameters are to be chosen by the practitioner to give the precise cost-benefit function for the intended application. Thus our analysis is applicable to any real-world system whose cost-benefit curve takes a similar shape. These will be combined with the penalty functions in Section 4 to provide three composite measures.

### 3.1 The aircraft routing problem

As airlines seek to maximize profit, the turn-around time for aircraft at airports becomes increasingly short, so as maximize the number of flights performed by aircraft in a given flight rotation. One of the major drawbacks of this approach is that unexpected disruptions can cause a series of knock-on, reactionary delays, affecting not only the schedule of a given aircraft, but those of other airlines [34]. Importantly, the departure time punctuality of aircraft is directly related to the arrival punctuality of inbound aircraft [35]. As noted in [36], arrival delays associated with inbound aircraft not only consume the scheduled turnaround time of an aircraft, but also disturb plans at an airport leading to longer aircraft ground service times than those scheduled.

Improving the punctuality of services may lead to significant savings through better utilization of an airline’s resources. For example, the cost of one minute of strategic buffer for an A320 is estimated at €49. An increase in punctuality that leads to a saving of 5 minutes of delay for 50% of schedules, would be worth approximately €1 billion per annum [34].

We now outline three possible cost-benefit scenarios for different aircraft arrivals at an airport. We examine three possible scenarios in which different aircraft type, mode (passenger/freight) and time of day affect the cost-benefit, and cost penalty for the airline. We denote these three different arrival scenarios by Scenario 1, 2 and 3 respectively. The main objective is to examine the cost benefit and punctuality improvement, associated with decreasing the average arrival time for each scenario. In the figures that follow, increases in time correspond to greater reductions in transit-time.

#### 3.1.1 Scenario 1: Long-Haul Passenger Service

An aircraft is considered to have arrived “on time” if it arrives within 15 minutes of its scheduled arrival time. Thus, any improvement in average arrival time will lead to a reduction in knock-on delays and penalties for missing slot times. An improvement in punctuality will ensure passengers have a greater chance of connecting with outbound flights, crew have sufficient time to change aircraft if operating multiple sectors and reduces the incidence of last-minute gate changes with possible lost passengers [34]. We assume a slowly-decreasing linear relationship, capturing the ability to achieve significant improvements without a significant increase in cost.

Decreasing the average arrival time by greater than 15 minutes may still be beneficial, as was noted by [37] who stated an emerging trend for U.S. flights which appear to be landing at destination airports earlier, relative to scheduled arrival times, allowing for an improvement to punctuality. However, adding too much buffer time to the schedule leads to a linear increase in opportunity costs when the duration of saved time becomes long enough for an aircraft to carry out an additional flight. Hence the airline schedule time cost is assumed to have a linear marginal cost function to account for this increasing opportunity cost [36].

**Fig 2:**
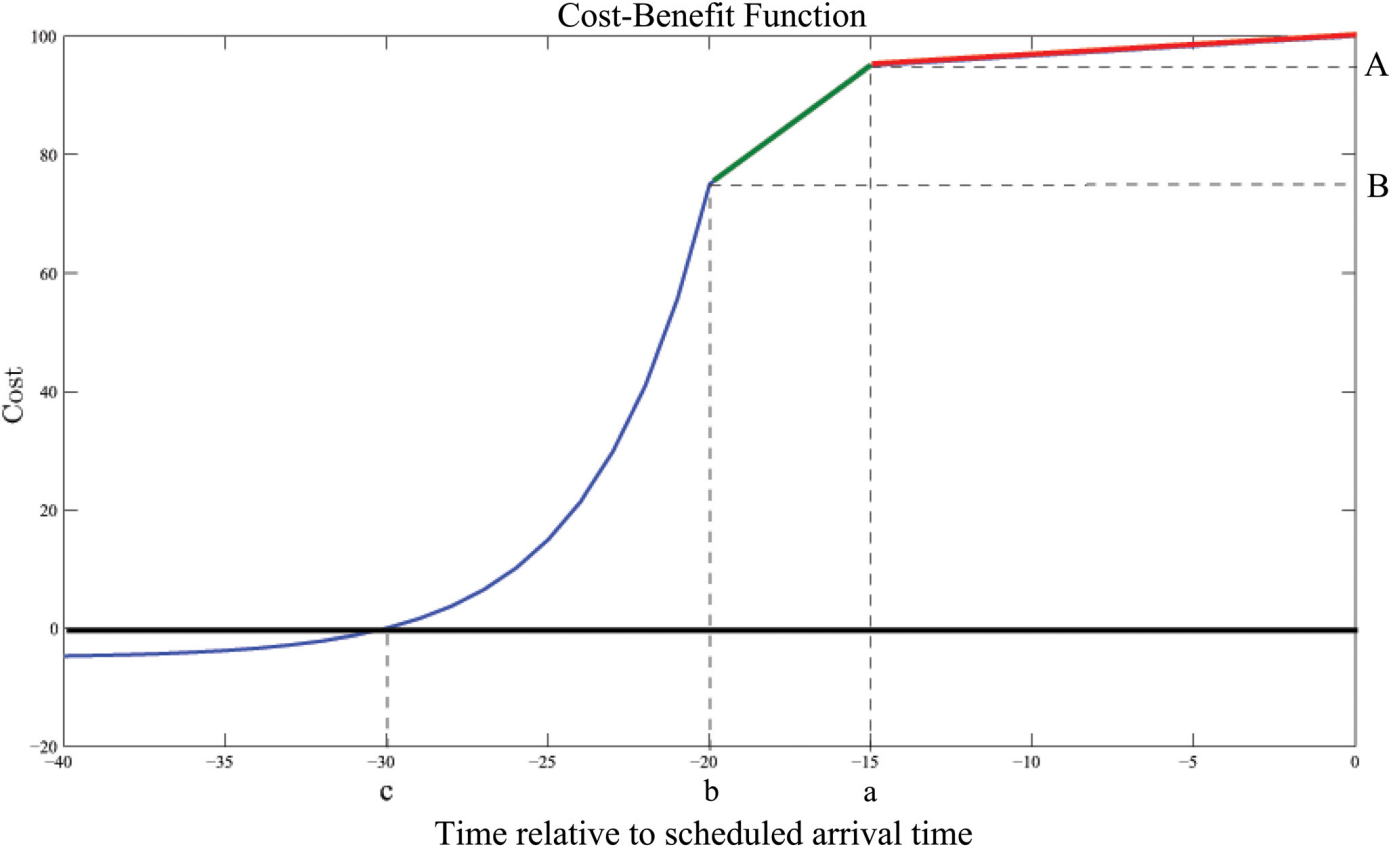
The Cost-Benefit Curve for Scenario 1.

A reduction of more than 20–30 minutes becomes exponentially less beneficial and eventually detrimental as a result of congestion of airspace, inaccessibility of runway and gate and fuel costs. Although passengers will generally appreciate the fact that more flights arrive (more than 15 minutes) ahead of schedule, this also brings more instability to the network (e.g. park and gate allocation at airports, staff planning for Ground Handling Agents, changed mix of departing and arriving traffic for ATC, etc). Additional analysis and study is necessary to determine the true impact of an increase in early arrivals [34]. We define the following possible general cost benefit-curve for Scenario 1 using Eq 8, depicted in Fig 2. Note that decreasing values of time correspond with the amount by which the average arrival time is decreased. For example, *t* = −5, corresponds to decreasing the average scheduled arrival time by 5 minutes.

$$\begin{array}{c} C\left(t\right)=\{\begin{array}{cc} \left(B+D\right)e^{-\left(-\frac{1}{c-b}\right)\ln\left(\frac{D}{B+D}\right)\left(t-b\right)}-D & t\leq b\\ (\frac{B-A}{b-a})\left(t-a\right)+A, & b<t\leq a\\ (\frac{A-100}{a})t+100, & a<t\leq 0 \end{array} \end{array}$$ (8)

#### 3.1.2 Scenario 2: Short-Haul Freight Service

**Fig 3:**
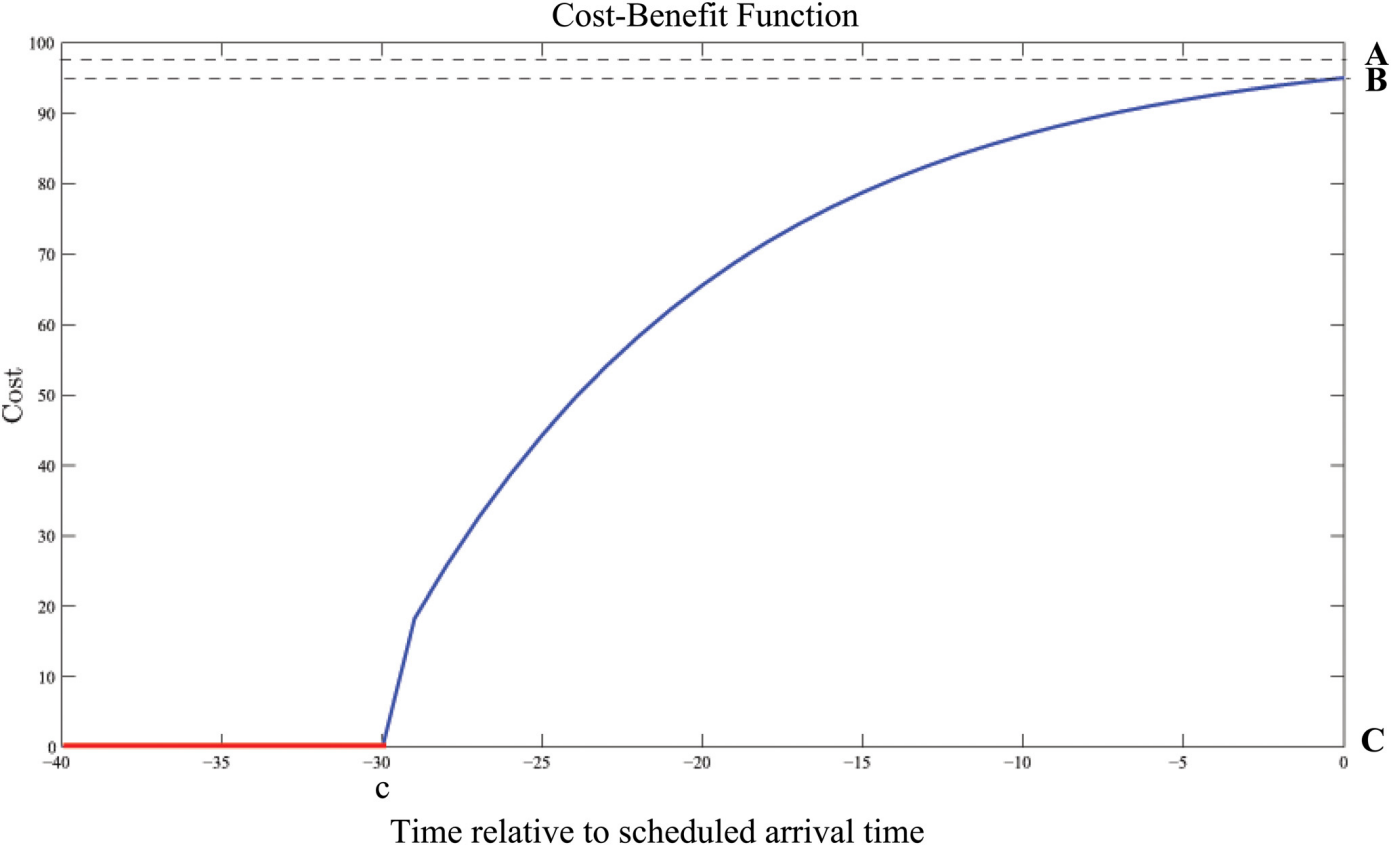
The Cost-Benefit Curve for Scenario 2.

Improving the average arrival time for a freight service is highly beneficial for both the service provider and the customer, as it reduces uncertainty for downstream customers in the supply chain. After a certain point, the benefits begin to decrease exponentially as a result of interactions with other passenger aircraft services sharing the same infrastructure (passenger services take priority over freight services), congestion and limits placed on approach speed, congestion and headway. Eventually these limitations provide an upper bound on the amount by which the average arrival time may be improved. At these speeds, there is no benefit to the operator for any additional increase in speed. We propose a possible general Cost-Benefit function for this short-haul freight scenario given in Eq 9, depicted in Fig 3.

$$\begin{array}{c} C\left(t\right)=\{\begin{array}{cc} 0, & t\leq c\\ A-\left(A-B\right)\exp(\frac{1}{c}[\ln(A-C)-\ln(A-B)]t), & c<t\leq 0 \end{array} \end{array}$$ (9)

#### 3.1.3 Scenario 3: Short-Haul Passenger Service

**Fig 4:**
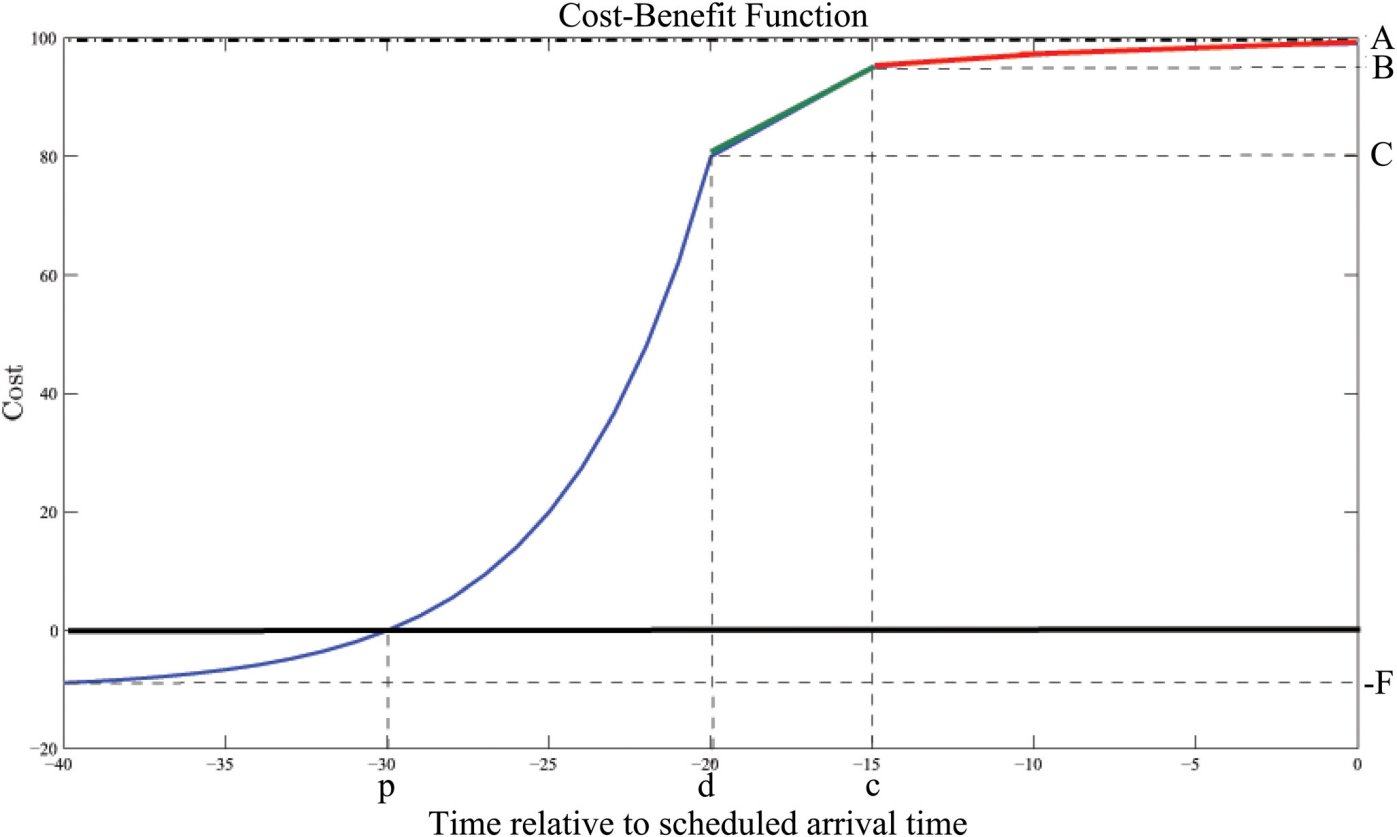
The Cost-Benefit Curve for Scenario 3.

This scenario is similar to that of Scenario 1, with the exception of using a slower rate of decrease in cost-benefit for minor adjustments to the average arrival time. This modification is intended to capture the fact that short-haul aircraft typically requiring less headway for landing than long-haul flights, and thus impose less of an impact in terms of increased congestion at airport facilities, for reasonably minor decreases in average time. The equation of the general cost-benefit curve pictured above may be defined in a piecewise manner as in Eq 10, and is depicted in Fig 4.

$$\begin{array}{c} C\left(t\right)=\{\begin{array}{cc} \left(C+F\right)e^{-\left(-\frac{1}{p-d}\right)\ln\left(\frac{F}{C+F}\right)\left(t-d\right)}-F & t\leq d\\ (\frac{C-B}{d-c})\left(t-c\right)+B, & d<t\leq c\\ A-\exp(\frac{\ln(A-B)t}{c}), & c<t\leq 0 \end{array} \end{array}$$ (10)

## 4 Penalty Function

In this section we define a penalty function that penalizes negative deviations (delays) from the scheduled arrival time. This measure will be combined with the cost-benefit functions defined in the three scenarios of the previous section so as to arrive at a composite benefit function *B*(*t*), for each of these three scenarios.

As we defined earlier, a transport service is considered punctual if the service arrives earlier than the punctual time *t*_*p*_, promised to the customers. If the service is delayed so that the transit time is greater than *t*_*p*_, a penalty cost for late arrival is incurred by the service provider [38]. We refer to the delayed costs as the costs associated with the delayed arrival, when compared with the time for punctual service.

Delays in service operational time can induce dramatic down-the-line effects for specific transit routes, and can even propagate network-wide. These delays only affect the transport provider, forcing them to provide alternative services and pay penalty costs upon late arrival. Moreover, passenger good-will is also affected. The delay is a major concern for the service actors in different transport services such as rail freight delivery [39], bus and rail transportation [40], ocean container and passenger shipping [41], and air transportation [42]. For example, the delays in the European airport transportation has been the major concern for the industry and a relentless source of complaints from the passengers while the delay-associated costs were approximately €6.6 billion in 1999 and €1.3 billion in 2007 [42].

We now propose a penalty function to reflect the cost of delay. For the time window $t\in [t_{min},t_{p}]$ promised to customers, there are no penalty costs associated with the delay in operation time. When the transport service is delayed, that is when $t\in (t_{p},t_{max}]$, the service provider must pay the penalty for the delay, with the penalty cost typically scaling as a function of the number of delay minutes as in Eq 11 [43].

$$\begin{array}{c} P\left(t\right)=\{\begin{array}{cc} 0, & t\in [p_{min},p_{s}]\\ H(t-p_{s}), & t\in [p_{s},p_{max}] \end{array} \end{array}$$ (11)

Combining this delay cost measure with the cost-benefit measure, we arrive at a new composite measure *B*(*t*) defined as follows:

$$\begin{array}{c} B(t)=C(t)-P(t). \end{array}$$ (12)

This new composite measure captures the cost-benefit for costs associated with both positive and negative deviations from the schedule.

In this paper, we generate three different composite cost-benefit measures *B*_1_(*t*), *B*_2_(*t*) and *B*_3_(*t*) and compare their ability to more accurately predict total cost-benefit with a simple linear function used in the literature.

## 5 Solution Approach

Using the new composite measure *B*(*t*), we wish to determine the optimal choice of average arrival time denoted by ‘*t*_*a*_’, so as to increase the level of service punctuality, whist simultaneously ensuring a minimum level of cost-benefit to the service operator. These two competing objectives naturally result in a multi-objective optimization problem [44]. However, as cost-benefit is measured in cost units, and punctuality of service in a probabilistic sense (as a percentage), it is not clear how to incorporate both of these quantities into a single optimization objective. Intuitively, it is natural to expect the objectives of the service operator would be to increase punctuality whilst minimizing the total variation to the original schedule. In particular, slot-times for the arrival of aircraft usually do not vary significantly from year to year, owing to the expense involved with bidding and purchasing new or alternative slots. However, it is also important for airlines to ensure that a certain percentage of total flights consistently arrive within their on-time performance window, so as to ensure that they retain their particular slot time.

In order to capture these aspects, we propose to maximize the average arrival time *t*_*a*_, subject to the constraints that the punctuality level *G*(*t*∣*λ*_*i*_), must be above a certain percentage threshold *α*, and the Cost-Benefit above a given cost *β*.

$$\begin{array}{ccc} \text{Maximize:} & & \;t_{a}\\ \text{Subject}\;\text{to:} & & \;G(t|\lambda_{i})\geq \alpha \\ & & \;\;B(t)\geq \beta \end{array}$$ (5)

where *α* ∈ {0,1} is a pre-defined level of punctuality of service, and *β* ∈ ℝ, the cost-benefit.

## 6 Empirical Results

To investigate the effectiveness of this new composite measure in providing improved punctuality, we analyze the improvement achieved via the use of these composite measures over the standard linear functional.

We take for our example, a long-haul passenger flight arriving at a busy international airport. We assume the average transit-time of 10.5 hours, with a scheduled arrival time *t*_*s*_ of 10 hours. As previously mentioned, a passenger aircraft service is considered to be on time if it arrives within a 15-minute time window of the scheduled arrival time. Since this airline wishes to retain their arrival slot, this flight is an obvious candidate for improvement, as improving the average arrival time by 15 minutes (so as to fall within this window). This will result in a greater number of flights being classified as on time. To develop the probability function for the reduced transit-time scenario, we assume the following support for arrival time: *t* ∈ [−15,30]. In this scenario we set specific values for each of the parameters so as to model a possible cost-benefit scenario for an airline. This will be referred to as Scenario 1. We similarly choose parameter values for Scenario 2 and 3, so as to obtain a comparison for different cost-benefit functional choices.

The Cost, Penalty and Composite Cost-Benefit functions from each Scenario used in this analysis are listed below.

- **Scenario 1**:
  $$\begin{array}{c} C_{1}\left(t\right)=\{\begin{array}{cc} 80e^{-\frac{1}{10}\ln\left(16t+320\right)}, & \;t\leq -20\\ 4t+155, & -20<t\leq -15\\ \frac{1}{3}t+100, & -15<t\leq 0 \end{array} \end{array}$$ (14)
  with penalty function *P*_1_(*t*) = *t* ^2^, resulting in the Cost-Benefit Function *B*_1_(*t*) = *C*_1_(*t*)−*P*_1_(*t*).
- **Scenario 2**:
  $$\begin{array}{c} C_{2}\left(t\right)=\{\begin{array}{cc} 0, & \;t\leq -15\\ 100-5e^{-\frac{1}{15}\left(\ln\left(18\right)t\right)}, & -15<t\leq 0 \end{array} \end{array}$$ (15)
  with penalty function *P*_2_(*t*) = *t* ^2^, resulting in the Cost-Benefit Function *B*_2_(*t*) = *C*_2_(*t*)−*P*_2_(*t*)
- **Scenario 3**:
  $$\begin{array}{c} C_{3}\left(t\right)=\{\begin{array}{cc} 100-\exp(-\frac{\ln(5)t}{15}) & \;t\leq -20\\ 3t+110, & -20<t\leq -15\\ 90\exp(-\frac{1}{10}\ln\left(\frac{1}{9}\right)\left(t+20\right))-10, & -15<t\leq 0 \end{array} \end{array}$$ (16)
  with penalty function $P_{3}(t)=e^{\frac{1}{4}t}$, resulting in the Cost-Benefit Function *B*_3_(*t*) = *C*_3_(*t*)−*P*_3_(*t*)

We compare each of these scenarios with the following standard functional.

- **Linear Function**:
  $$\begin{array}{c} C_{L}\left(t\right)=\{\begin{array}{cc} t+100, & t<0 \end{array} \end{array}$$ (17)
  with penalty function *P*_*L*_(*t*) = *t*, resulting in the Cost-Benefit Function *B*_*L*_(*t*) = *C*_*L*_(*t*)−*P*_*L*_(*t*).

**Fig 5:**
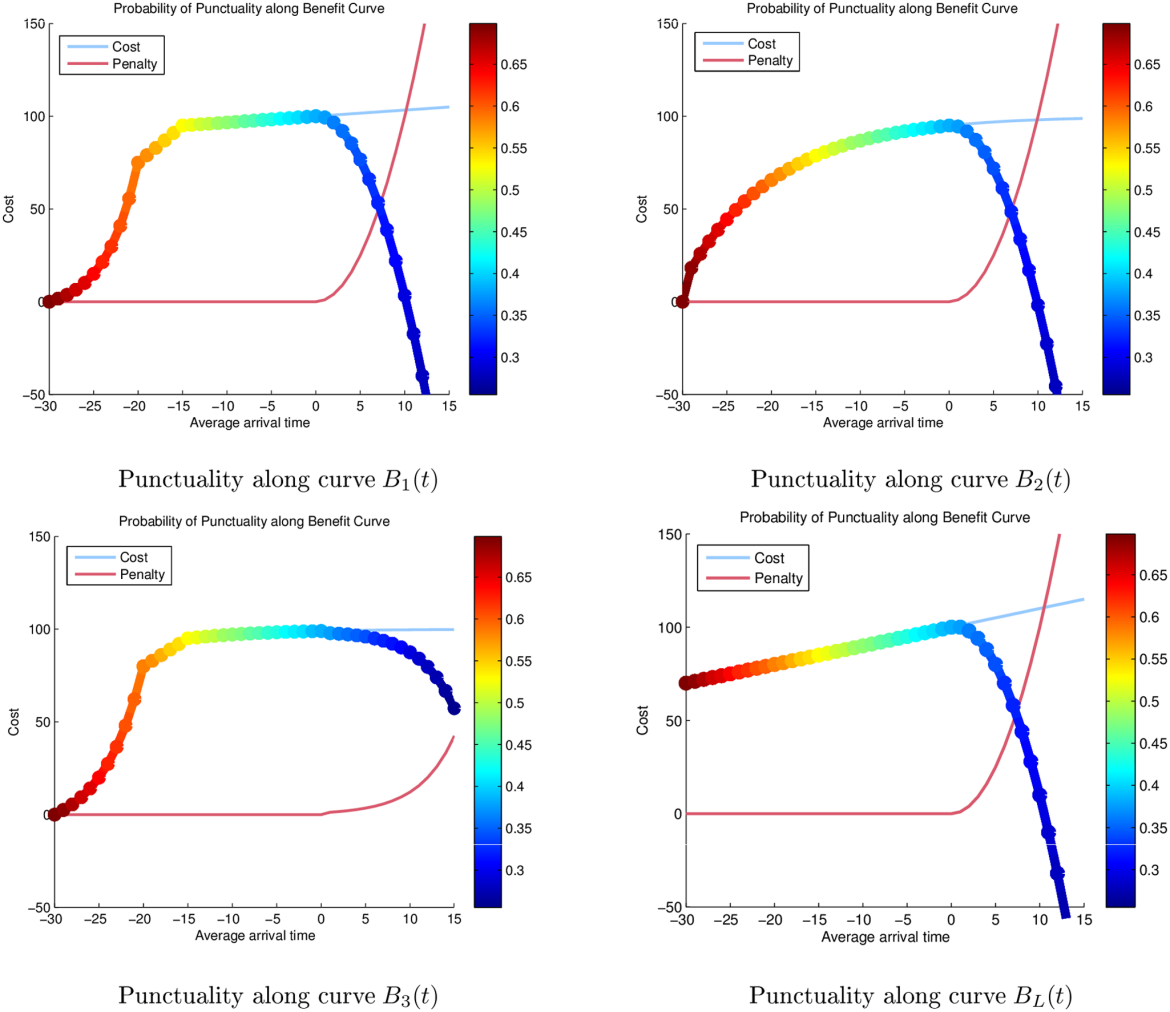
Punctuality vs cost-benefit and penalty function.

Fig 5 presents the probability of punctuality of a transport service vs cost-benefit and penalty function for the cases discussed above.

It may be observed from the figures above, that setting the level of punctuality to *α* = 0.55, and lower bound on Cost-Benefit to *β* = 50, we achieve the solutions presented in Table 1.

**Table 1:** Optimal arrival time with the corresponding cost for each benefit function.

**Benefit Curve**	**Optimal *t*_*a*_**	**Cost Units**
*B*_1_(*t*)	-15	95
*B*_2_(*t*)	-15	79
*B*_3_(*t*)	-15	95
*B*_*L*_(*t*)	-30	70

It may be observed that this choice of parameters yields results for Scenario 1, 2 and 3 that ensure that the specified flight will arrive on-time, with a 55% probability, for a reduction of only 5–21 cost units in cost-benefit (profit); in addition to minimizing the knock-on effects of delay. This is to be contrasted with the result for Scenario 1 in which the delay cost of arriving > 15 minutes late incurred a cost penalty of > 50 cost units. That is, the cost of delay may be in some cases almost more than twice as expensive than that of a decrease in transit-time. This is an important result, as it is often perceived by transport planners, that the cost of increasing punctuality results in a greater decrease to profit than a late arrival.

Additionally for the same set of parameters, the linear functional denoted by *B*_*L*_(*t*) determines that an adjustment of 30 minutes to the schedule will yield the best results. However, this solution is most likely unattainable in practice, and unlikely to be used by a real-world airline, as it would entail encroaching on the slot time of another aircraft. Moreover, as the linear functional ignores the effects such as increased fuel cost for faster transit-times, the cost-benefit would be over-estimated for such a choice of arrival time. This further underscores the need for cost-benefit functions to reflect the individual form of transport.

## 7 Discussion

With the rapid development in product globalization and just-in-time production over the last two decades, area-specific, reliable, responsive and customer-oriented transport services are currently in high demand and are becoming of increasing importance. Identifying and obtaining an understanding of underlying transit-factors so as to achieve a quality evaluation of transport services is a key challenge for both short-term and long-term regional and metropolitan freight mobility management and planning. Moreover, this is crucial within the context of a competitive transport market.

Among the fundamental attributes of transport services, transit time and punctuality are of utmost importance as they are invariably correlated, according to the specific network structure. However, adjustments to expected transit-time usually incur additional operating costs. It is therefore desirable for the operator during the planning stage, to obtain an insight into the effect of proposed changes to the schedule on both reliability and operating cost. Moreover, striking the correct balance between punctuality improvement and cost minimization is of particular importance.

Having a more complete understanding of underlying factors in the evaluation of the quality of transport services is a key challenge in the short-term and long-term regional and metropolitan freight and passenger mobility planning, particularly with a competitive transportation market all around the world. With the objective of quantitatively evaluating the trade-offs between transit time and reliability, as two influential transport service quality attributes, this paper provides insights on how managing the time-based transport attributes create a competitive advantage for both customers. Using a statistical probability function, a methodology is developed in this paper for calculating the probability of reliability for a certain transport service under different cost-benefit scenarios. Using these scenarios we observed how changes to the average arrival time can affect the probability of punctuality and the associated service costs and/or benefits.

## Supporting Information

S1 Supplementary File Simulation Data. The data used in the simulation presented in this study.
(XLSX)

S2 Supplementary File Codes. The codes used for the simulation presented in this study.
(M) Click here for additional data file.
